# Biocontrol and plant growth promotion by combined *Bacillus* spp. inoculation affecting pathogen and AMF communities in the wheat rhizosphere at low salt stress conditions

**DOI:** 10.3389/fpls.2022.1043171

**Published:** 2022-12-08

**Authors:** Chao Ji, Zhizhang Chen, Xuehua Kong, Zhiwen Xin, Fujin Sun, Jiahao Xing, Chunyu Li, Kun Li, Zengwen Liang, Hui Cao

**Affiliations:** ^1^ College of Seed and Facility Agricultural Engineering, Weifang University, Weifang, Shandong, China; ^2^ Key Laboratory of Biochemistry and Molecular Biology in University of Shandong Province, Weifang University, Weifang, Shandong, China; ^3^ Shandong Yongsheng Agricultural Development Co., Ltd. Yongsheng (Shouguang) Vegetable Technology Research Institute Co., Ltd, Shandong Engineering Research Center, Weifang, Shandong, China; ^4^ Runxin Fruit and Vegetable Cultivation Cooperative of Weifang Economic Development Zone, Weifang Agricultural Bureau, Weifang, Shandong, China; ^5^ College of Foreign Languages, Weifang University, Weifang, Shandong, China; ^6^ Weifang Hanting Vestibule School, Weifang Education Bureau, Weifang, Shandong, China; ^7^ College of Forestry, Shandong Agriculture University, Taian, Shandong, China; ^8^ Taishan Forest Ecosystem Research Station, Key Laboratory of State Forestry Administration for Silviculture of the Lower Yellow River, Shandong Agricultural University, Taian, Shandong, China

**Keywords:** salt stress, microbial inoculants, plant growth-promoting rhizobacteria, arbuscular mycorrhizal fungi, community structure, wheat yield, disease control

## Abstract

Applying plant growth-promoting rhizobacteria (PGPR) improves the efficiency of soil-borne disease control and is considered a sustainable practice. However, the effect of PGPR on the fungal community, especially pathogenic fungi and arbuscular mycorrhizal fungi (AMF), remains unclear. In this study, we examined the effects of a compound microbial agent (consisting of *Bacillus subtilis* HG-15 and *Bacillus velezensis* JC-K3) on the incidence and yield of wheat under low salt stress, as well as compared the diversity and community composition of the rhizosphere fungal and AMF communities of wheat in the CK (not inoculated bacterial agent) and BIO (inoculated with a bacterial agent) groups. Chlorophyll relative content (SPAD), net photosynthesis rate (*Pn*), transpiration rate (*Tr*), leaf water use efficiency (*WUE*_L_), grains per spike and wheat yield in the BIO group increased more than in the CK group. The number of diseased plants and disease incidence was observed to be reduced. The relative efficacy reached 79.80%. We classified 1007 fungal operational taxonomic units (OTU) based on Miseq sequencing data: 11 phyla, 173 families, 319 genera, and 521 species. Fifty-four OTUs were classified from the AMF effective sequences, including 1 phylum, 3 families, 3 genera, and 17 species. The inoculation of bacterial agents reduced the relative abundance of pathogen genera such as *Gibberella*, *Fusarium*, *Cladosporium*, and *Alternaria* in wheat rhizosphere. It increased the relative abundance of AMF species such as Glomus-group-B-Glomus-lamellosu-VTX00193, Glomus-viscosum-VTX00063, and Glomus-Glo2-VTX00280. In addition, pH, EC, exchangeable K, available N, total N, organic matter, and olsen P were the main driving forces for shaping wheat rhizosphere fungi. The pH value was positively correlated with the relative abundance of fungal communities in soil, especially *Gibberella*, *Cladosporium*, *Fusarium*, and *Alternaria*. In summary, inoculation with *Bacillus subtilis* HG-15 and *Bacillus velezensis* JC-K3 affected wheat yield, incidence, rhizosphere soil chemical properties, rhizosphere fungi, and AMF fungal diversity and community. The findings may provide a theoretical foundation and strain support for constructing efficient PGPR-community and clarifying its mechanism of pathogenic bacteria inhibition.

## Introduction

Salt stress is one of the major abiotic stresses limiting crop production in arid and semi-arid regions; salinity threatens at least 20% of the world’s cropland to varying degrees (Zhu et al., 2011). Salt stress causes osmotic, ion toxicity, and oxidative stress, disrupting the integrity of cell membrane systems and ultrastructure in photosynthetic systems. The structure and function of inter-root soil microbial communities are also susceptible to significant changes because of salt stress, directly affecting crop growth and yield (Munns and Tester, 2008). Although plants can produce some yield in saline soil habitats, most crops and tree species are not highly salt tolerant, with major food crops such as wheat, maize, rice, and barley suffering yield losses of up to 70% because of salt stress (Acquaah, 2007). With the intensification of population growth, land and food conflicts, and climate change, improving the soil environment and promoting crop yields in low salt-stressed soils are crucial for promoting economic development, ensuring food security, relieving population pressure, and maintaining ecological balance.

Crop diseases caused by soil-borne pathogens are another important constraint to high-quality, high-yielding food and sustainable agricultural development worldwide. The sexual stage of *Fusarium* is often *Gibberella*, which can cause crown rot, stem rot, and blast rot in wheat (Javad et al., 2006); more seriously, *Fusarium* can cause wilt and root rot in over 100 crops (Li et al., 2015), such as wheat wilt caused by *Fusarium graminearum* (Drakopoulos et al., 2020); and rice blight caused by *Fusarium moniliforme* (Alam et al., 2010). The *Gibberella*, on the other hand, causes fusarium head blight in wheat, barley, and other cereal crops (Oliver et al., 2008).

Salt-tolerant PGPR has probiotic properties, such as the production of phytohormones, siderophores, ACC deaminase (ACCD), and compatible solutes like proline, which directly reduce the level of plant stress hormone ethylene and are involved in the conversion and cycling of carbon (C), nitrogen (N), and phosphorus (P), as well as other substances and energy in the soil (Singh et al., 2015; Delgado-Baquerizo et al., 2016). These mechanisms are related to improving plant salt tolerance and induced system tolerance (IST), which can effectively reduce salt stress damage in plants, promote crop growth, and suppress pathogen proliferation (Etesami and Beattie, 2018). Arbuscular mycorrhizal fungi (AMF) can form symbiotic relationships with most crops. They can improve salt tolerance and disease resistance to soil-borne diseases by promoting plant growth, water uptake, and initiating host plant defense responses, besides improving soil nutrient acquisition (Cameron et al., 2013; Ruiz-Lozano et al., 2016; Despina et al., 2018; Bennett and Groten, 2022). Although AMF may not be “sustainable saviors” for agricultural intensification (Thirkell et al., 2017), AMF does have the potential to aid in the nutritional assimilation of grains (Thirkell et al., 2020). Most studies have focused on the pro-growth effects of exogenous inoculation of PGPR or AMF on host plants. In contrast, little research has been done on the synergistic effects of salt-tolerant PGPR with other microorganisms in the root zone on saline soils and crops. There is a specific lack of research on the response of AMF communities to salt-tolerant PGPR.

The rational application of microorganisms in modern agriculture can maximize yields while minimizing inputs under anticipated environmental perturbations (Vries et al., 2020). *Bacillus subtilis* HG-15 and *Bacillus velezensis* JC-K3, the strains used in this study, are salt-tolerant bacteria obtained, respectively, from the rhizoplane and inside the roots of wheat in the saline soil of Yellow River Delta; the strains did not inhibit each other and were able to colonize stably in the inter-root of saline wheat, both with the properties of producing ACC deaminase, IAA, Siderophore, and proline. The strains have been demonstrated to promote wheat seedling growth, reduce salt stress damage, and improve photosynthesis and osmoregulation in pot experiments (Ji et al., 2021; Ji et al., 2022). More studies have shown that combined bacterial inoculum has higher environmental adaptability, biological viability, and synergistic metabolism levels than single strains and has a greater impact on soil-plant material cycling (Dombrowski et al., 2018). Therefore, this study will investigate the effects of combined biocontrol agents composed of these two strains on fungi, especially the AMF community in wheat rhizosphere soil, as well as the relationship between microbial community response, wheat yield, and disease resistance. The results of this study may provide a theoretical foundation and data support to clarify further the mechanisms of PGPR and AMF promotion and disease prevention under salt stress, as well as a reference point for future research on how to mobilize indigenous AMF.

## Materials and methods

### Biocontrol strain and culture medium

The microbial inoculant is a compound agent of *Bacillus subtilis* HG-15 and *Bacillus velezensis* JC-K3. Both strains exhibit efficient antagonistic activity and other growth-promoting characteristics, as described in our previous studies (Ji et al., 2021; Ji et al., 2022). Luria-Bertani liquid medium was used as a seed and fermentation medium. When the spore formation rate in the fermentation liquid was greater than 95%, diatomite was sterilized at a high temperature (121 °C, 20 min) and added at a concentration of 10% to the fermented liquid. The bacteria were allowed to adsorb to the diatomite. The suspension was centrifuged at 3,100 × *g* for 20 min. The supernatant was removed, and the sediment was stored at −40 °C for 48 h before being placed in a lyophilizer (Labconco FreeZone^®^ Plus 4.5 L; Kansas City, MO, USA) and treated at −48 °C and 9 Pa for 48 h (Ji et al., 2020). The density of HG-15 and JC-K3 in the resultant solid microbial agent was 472 × 10^8^ CFU g^−1^ and 511 × 10^8^ CFU g^−1^, respectively. The bacterial preparations were mixed with sterile diatomite and diluted to 20 × 10^8^ CFU g^−1^.

### Experimental design

Between October 2021 and July 2022, an experimental plot system was established in the Weifang Changyi area of Shandong Province, China (119°31′55″E, 36°38′47″N). The soil in the experimental area was mildly salinized alluvial, and the surface soil texture was medium loam. The chemical properties of initial soil were pH 8.11, EC 316 μs cm^-1^, organic matter 23.51 g kg^-1^, total nitrogen 1.792 g kg^-1^, available nitrogen 79.35 mg kg^-1^, Olsen P 18.83 mg kg^-1^, and exchangeable potassium 97.06 mg kg^-1^. Nutrient Agar (NA) medium and Potato Dextrose Agar (PDA) medium were used to isolate and count culturable bacteria and fungi in rhizosphere soil, respectively. The number of culturable bacteria and fungi in soil was 5.73 × 10^4^ and 2.34 × 10^3^ CFU g^-1^ dry weight of soil, respectively. This study used a completely randomized block design with three replicates per treatment (CK and BIO), with each replicate consisting of an area of 40 m^2^ (8 m length × 5 m width) insulated with a buffer zone.

Wheat seeds (CV. *Jimai* 22) were surface-sterilized with 1% sodium hypochlorite for 5 min, washed 3–5 times with sterile water, and sown artificially on the plot at 0.6 kg per 40 m^2^ on October 12, 2021. At the jointing stage, wheat seedlings in the treatment groups were first irrigated with bacterial preparations (previously dissolved in water at 5.0 kg per 40 m^2^) on February 20, 2022, and then on March 08, 2022; wheat seedlings in the control group were irrigated with the same volume of tap water. Before replanting, a basal fertilizer (45% Yangfeng compound fertilizer, N14-P16-K15; 1.2 kg per 40 m^2^) was used; the nitrogen fertilizer (urea, 1.5 kg per 40 m^2^) was used during the green-up period.

### Rhizosphere soil sampling and analysis

The bulk of the soil at the root of the wheat was removed by gentle shaking, leaving only rhizosphere soil; and the soil that remained adhered to the roots was considered rhizosphere soil. The residual soil was collected from the roots using a sterile brush (Smalla et al., 1993). Soil samples for the determination of microbial communities were quick-frozen in liquid nitrogen and stored in a refrigerator at -80°C until they were extracted and analyzed. Soil samples for chemical determination were stored at 4 °C. Soil pH and EC values were analyzed using digital pH (FE20) and EC (FE930) meters (Mettler Toledo, Switzerland), respectively, with soil-water ratios of 1:2.5 and 1:5. The organic matter content in the soil was determined with a method described in Aj and Black (1934). Olsen P was determined using the method proposed by Olsen (1954). The total N was determined using the Bremner (2009) method. Available N and exchangeable potassium values were obtained using a method described in Jackson (1979).

### Chlorophyll relative content (SPAD) and photosynthetic parameters

Five flag leaves were randomly selected from each plot at 9:00–11:00 a.m. on 0, 10, 20, and 30 days after the flowering of winter wheat. The SPAD value was measured using a portable chlorophyll tester (SPAD 502, Minolta Camera Co., Ltd., Japan). Net photosynthetic rate (*Pn*) and transpiration rate (*Tr*) were measured using an LI-6800XT portable photosynthetic instrument (Li-COR, Lincoln, NE, USA). The measuring time, date, and leaf position were all consistent with the wheat SPAD value. The photon flux was set to 1,200 μmol m^−2^ s^−1^, the blade temperature to 25 °C, the humidity to 55%, and the flow rate to 500 μmol s^-1^. Each treatment consisted of five leaves. Leaf water use efficiency (*WUE*_L_) was calculated using the following equation:


$$WUE_{L}=Pn/Tr$$

### Effects of microbial inoculants on disease

At the end of grouting, the main disease characteristics of white heads in wheat were investigated. Each treatment had three replicates, and each replicate had three sampling sites chosen at random. A total of 100 wheat seedlings were surveyed for each sampling point. The number of white spikes was counted, and the relative biocontrol effect was calculated using the following formula (Moussa et al., 2013).


$$\text{Disease incidence}=\frac{\text{The number of diseased plants}}{\text{Total number of wheat seedling}}\times 100\%$$

$$\text{Relative efficacy}=\frac{\text{Disease incidence in control}-\text{disease incidence in treatment}}{\text{Disease incidence in control}}\times 100\%$$

### Grain filling rate and yield

Ten wheat spikes were randomly collected from each plot at 7, 14, 21, 28, and 35 days after flowering. They were placed in the oven at 105°C for 30 min and dried to constant weight at 75°C for 72 h. The filling rate was calculated as grain weight/filling duration (Deng et al., 2021). For wheat maturity, each plot randomly selected 1 m^2^ to investigate the number of spikes per unit area, selected 100 spikes to investigate the number of grains per spike, harvested 4 m^2^ threshing, and weighed after natural air drying (grain moisture content is approximately 12.5%), calculated yield per unit area, and investigated 1000-grain weight. The procedure was repeated five times for each treatment.

### DNA preparation and polymerase chain reaction-based amplification

Soil genomic DNA was extracted from 0.5 g soils with a FastDNA SPIN Kit for Soil (MP, California, USA) according to the manufacturer’s instructions. The DNA quality was examined using 1.0% agarose gel electrophoresis, and the DNA concentration was quantified using a NanoDrop 2000 UV–Vis spectrophotometer (Wilmington, USA) (Zeng et al., 2021).

The fungal rDNA-ITS gene was amplified from the total soil genomic DNA using primers ITS1F (5′-barcode-CTTGGTCATTTAGAGGAAGTAA-3′)/2043R (5′-GCTGCGTTCTTCATCGATGC-3′). PCR was performed in triplicates in a 20-μL reaction tube containing 4 μL of 5× FastPfu Buffer, 2 μL of 2.5 mmol dNTPs, 0.8 μL of each primer (5 μmoL), 0.4 μL FastPfu Polymerase, 10 ng template DNA, and adding ddH_2_O to a final volume of 20 μL.

Nested PCR was conducted to amplify fragments of AMF 18S rRNA gene with high specific amplification. The first PCR step was conducted in a 20 μL reaction tube containing 1 μL of genomic DNA (approximately 10 ng), 2 μL of 2.5 mM dNTPs, 0.4 μL of FastPfu DNA Polymerase (5 U μL^-1^), 0.4 μL of each primer (10 μM; AML1 (5′-ATCAACTTTCGATGGTAGGATAGA-3′)/AML2 (5′-GAACCCAAACACTTTGGTTTCC-3′) primer pair), 4 μL of 5-fold Fastpfu DNA Buffer (Takara, Dalian, China), and molecular-grade water. In the second PCR amplification, the products of first PCR step (with approximately 10 ng used as the template) were amplified in a 50-μL reaction tube with the primers AMV4.5NF (5′-AAGCTCGTAGTTGAATTTCG-3′) and AMDGR (5′-CCCAACTATCCCTATTAATCAT-3′), as described for the first PCR step. The thermal cycling conditions for both PCR steps were as follows: initial denaturation at 95 °C for 3 min; 27 cycles of 30 s of denaturation at 95 °C, 30 s of annealing at 55 °C, and 45 s of elongation at 72 °C; and final elongation at 72 °C for 10 min. PCR products were extracted using 2% agarose gels and purified using an AxyPrep DNA Gel Extraction Kit (Axygen, USA) according to manufacturer’s protocol and quantified using a QuantiFluor ST instrument (Promega, USA).

### Illumina MiSeq and bioinformatics analyses

Qualified and purified PCR products were sent to Majorbio BioPharm Technology Co., Ltd. (Shanghai, China) for sequencing on an Illumina MiSeq PE300 instrument (San Diego, USA). The raw sequences were deposited in NCBI Sequence Read Archive (SRA) database (accession number PRJNA869482). The forward and reverse raw sequences were merged using FLASH (Mago and Salzberg, 2011) by overlapping paired-end reads using a required overlap length of >10 base pairs (bp) and quality-controlled using Trimmomatic software (Bolger et al., 2014); low-quality sequences (average quality score< 20) containing ambiguous bases, sequences with no valid primer sequence or barcode sequence, and sequences with a read length< 50 bp were excluded. Moreover, the permitted maximum error ratio of overlapping sequences was 0.2, which was established as the basis for screening overlapping sequences.

After the sequences were merged and subjected to quality control, non-repeating sequences were extracted, and individual sequences that did not repeat were removed using Usearch 7.0 (Edgar, 2013); the sequences were subsequently clustered into operational taxonomic units (OTUs) with a 97% similarity cut-off using QIIME software (Caporaso et al., 2010). After the sequences were clustered, the taxonomy of each OTU was classified from the domain level to the OTU level using RDP Classifier algorithm against the MaarjAM database (Maarjam 081) (ÖPik et al., 2010), with a default confidence threshold of 0.7.

### Statistical analyses

Data analysis was performed using IBM SPSS 19.0. Plant and soil parameters followed a normal distribution. A student’s t-test and one-way ANOVA were used for parameter differences among plant parameters (*P*< 0.05). Redundancy analysis (RDA) was performed to examine the relationships between the relative abundance of fungal, AMF taxa and the chemical properties of soil samples by using Canoco 4.5.1 (Microcomputer Power, Ithaca, USA) software. The non-parametric factorial Kruskal-Wallis (KW) sum-rank test of the LEfSe software was used to detect the characteristics of significant abundance differences, and the groups with significant differences in abundance were found. LEfSe uses linear discriminant analysis (LDA) to estimate the effect of each component (species) abundance on the difference.

## Results

The soil’s nutritional status was analyzed for the plant growth study. To evaluate the effect of microbial agents, we performed a student’s t-test (*P*< 0.05). The EC (5.50%), exchangeable potassium (10.24%), Olsen P (5.68%), total N (7.22%), available N (10.16%), and organic matter (12.31%) of wheat rhizosphere soil treated with BIO were significantly higher than those treated with CK group (*P*< 0.05). The pH of CK group was significantly higher than that of the BIO treatment (2.04%, *P*< 0.05) **(** **Table 1** **)**.

**Table 1:** Effects of the inoculation with combined PGPR inoculation (BIO) compared to control (CK) on the chemical properties of wheat rhizosphere soil.

Treatment	EC(μs/cm)	Exchangeable K(mg/kg)	Olsen-P(mg/kg)	Total N(g/kg)	Available N(mg/kg)	Organic matter(g/kg)	pH
CK	342.0 ± 2.92b	101.52 ± 1.88b	18.49 ± 0.15b	1.94 ± 0.07b	85.41 ± 2.40b	26.49 ± 0.59b	7.99 ± 0.06a
BIO	360.8 ± 4.38a	111.92 ± 2.67a	19.54 ± 0.23a	2.08 ± 0.06a	94.09 ± 1.58a	29.75 ± 0.99a	7.83 ± 0.03b

Data are means ± standard deviation (SD) (n = 5). The small letters in the table represent the significant difference between the indexes of uninoculated and inoculated compound microbial agent wheat, P < 0.05.

**Figure 1:**
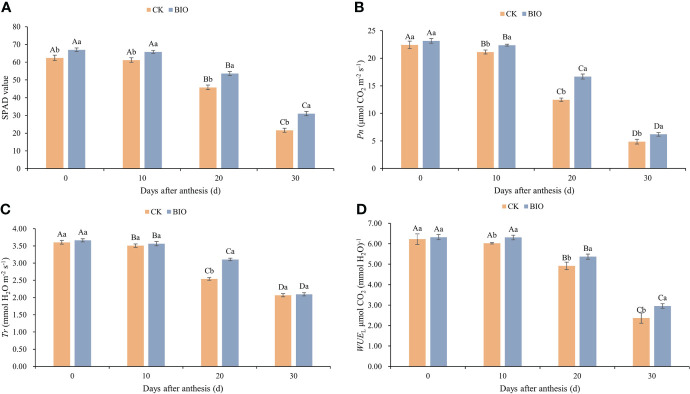
Effects of compound microbial agents on SPAD and photosynthetic parameters in flag leaf. CK and BIO represent the control and combined biocontrol agents inoculated plants, respectively. **(A)** SPAD: Chlorophyll relative content. **(B)** Pn: Net photosynthesis rate. **(C)** Tr: Transpiration rate. **(D)** WUEL: Leaf water use efficiency. Capital letters indicated significant differences between groups (one-way ANOVA, P < 0:05), whereas small letters indicate significant differences among control (CK) and compound microbial agents inoculated (BIO) plants at the same time point (student’s t-test, P < 0.05), respectively. The smaller bars are standard errors.

The SPAD content of wheat after flowering decreased as the growth process progressed in both treatments. Under the same treatment, no significant difference was observed in the SPAD content of wheat after flowering between days 0 and 10, but it decreased significantly on days 20 and 30 (*P*< 0.05). At each time point, the SPAD content of BIO treatment was significantly higher than that of the CK treatment (*P*< 0.05) (**Figure 1A**). With the advancement of growth process, *Pn* and *Tr* decreased significantly (*P*< 0.05). On days 10, 20, and 30, the *Pn* of the BIO treatment was significantly higher than that of the CK treatment (*P*< 0.05). On day 20, the *Tr* of the BIO treatment was significantly higher than that of the CK treatment (*P*< 0.05) (**Figures 1B, C**). Under both treatments, *WUE*_L_ decreased significantly on days 20 and 30 (*P*< 0.05). On days 10, 20, and 30, the *WUE*_L_ of the BIO treatment was significantly higher than that of the CK treatment (**Figure 1D**).

**Figure 2:**
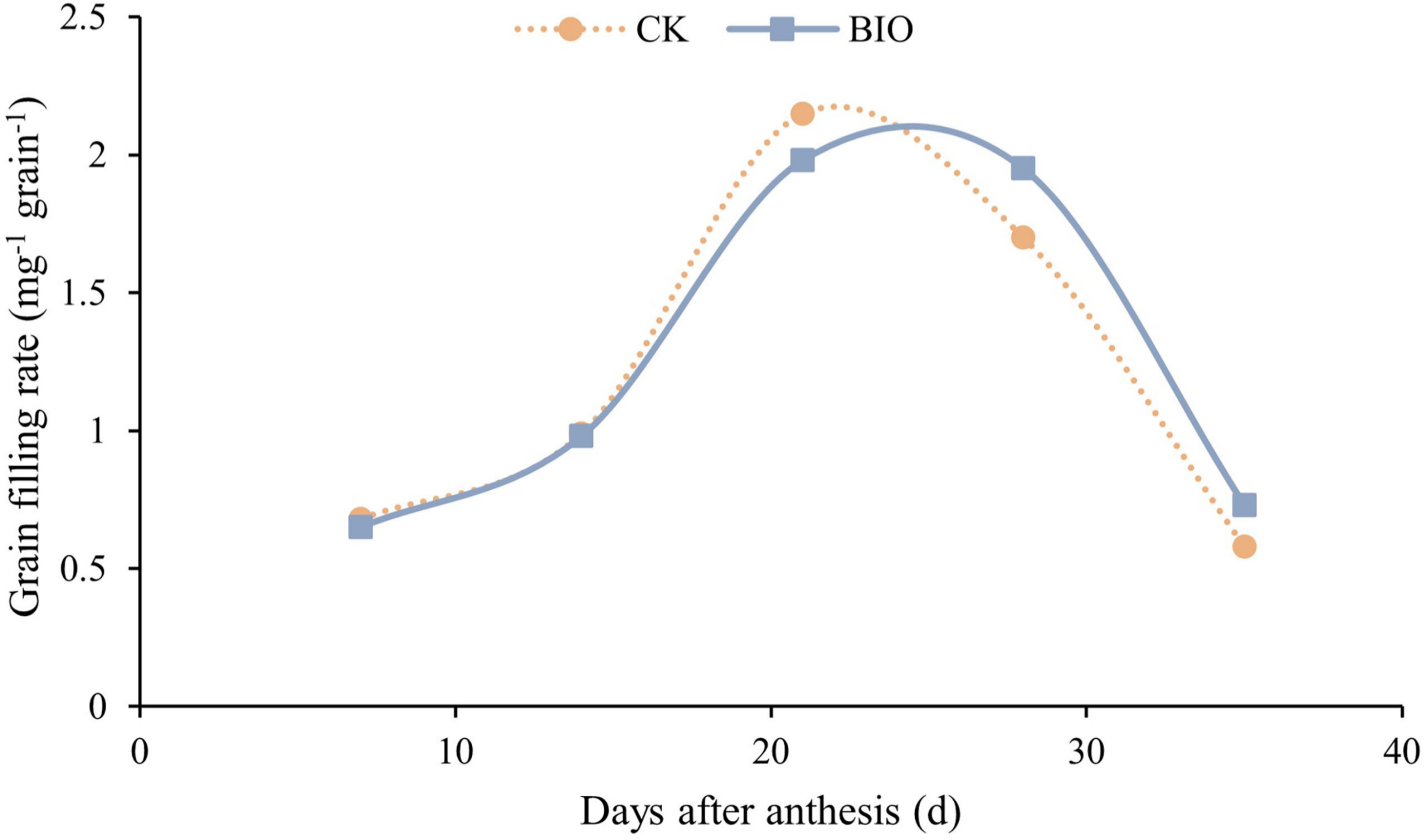
Effects of compound microbial agents on grain filling rate. CK and BIO represent the control and combined biocontrol agents inoculated plants, respectively.

The fitting curve (**Figure 2**) shows that the grain filling rate of CK group reached peaked on day 20 after flowering. The BIO group peaked on day 25 after flowering and was higher than the CK treatment during 30–35 days after flowering. The incidence of wheat disease was determined at the end of grain filling. Diseased wheat plants (79.24%) and disease incidence (79.84%) in the BIO group were significantly lower than those in the CK group (*P*< 0.05). The relative efficacy was 79.80% (**Table 2**). Inoculation with compound bacteria did not significantly change the spike number or thousand-grain weight of wheat. However, the grain number per spike (10.43%) and yield (8.77%) of the BIO group were significantly higher than those of the CK group (*P*< 0.05) (**Table 3**).

**Table 2:** Disease prevention efficacy by microbial inoculants.

Treatment	Total number of wheat seedlings	Diseased plants	Disease incidence	Relative efficacy
CK	300	79 ± 5.3a	26.30%a	—
BIO	300	16 ± 5.1b	5.30%b	79.80%

Data are means ± standard deviation (SD) (n = 5). CK and BIO represent the control and combined biocontrol agents inoculated plants, respectively. The small letters in the table represent the significant difference between the indexes of uninoculated and inoculated compound microbial agent wheat, P < 0.05.

**Table 3:** Effects of different treatments on grain yield and yield components in winter wheat.

Treatment	Spike number(×10^4^ hm^-2^)	Grain number per spike	1000-grain weight(g)	Yield(kg hm^-2^)
CK	653.14 ± 6.72a	34.53 ± 2.31b	42.17 ± 3.41a	7469.37 ± 357.6b
BIO	657.62 ± 6.84a	38.13 ± 2.06a	42.94 ± 2.11a	8124.52 ± 277.8a

Data are means ± standard deviation (SD) (n = 5). CK and BIO represent the control and combined biocontrol agents inoculated plants, respectively. The small letters in the table represent the significant difference between the indexes of uninoculated and inoculated compound microbial agent wheat, P < 0.05.

**Figure 3:**
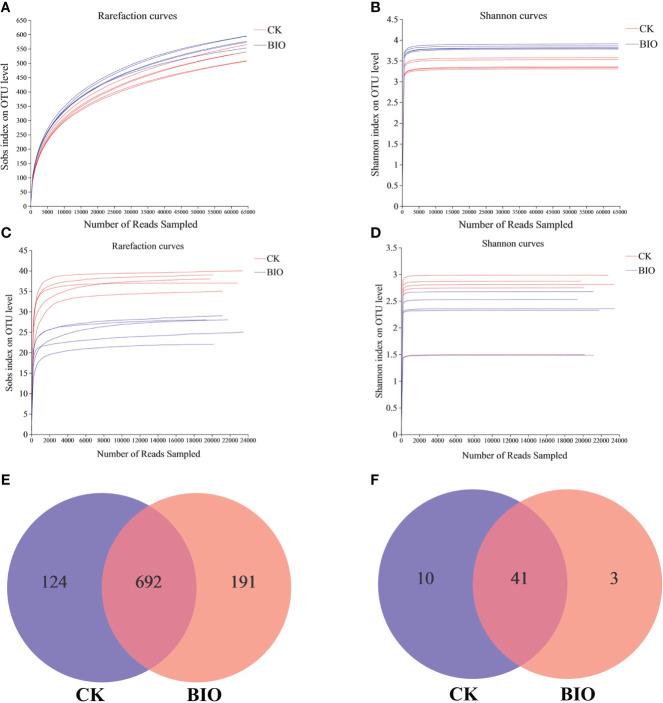
Different treatment samples generate microbial rarefaction curves, Shannon curves, and venn diagrams based on OTU level. **(A)** Fungi rarefaction curves; **(B)** fungi Shannon curves. **(C)** AMF rarefaction curves; **(D)** AMF Shannon curves. **(E)** The unique and shared fungal OTUs in CK and BIO groups; **(F)** the unique and shared AMF OTUs in CK and BIO groups. CK and BIO represent the control and combined biocontrol agents inoculated plants, respectively.

Wheat rhizosphere soil samples of the CK and BIO groups were analyzed to determine their community structure. A total of 759029 effective ITS sequences and 254269 effective AMF sequences were obtained, which accounted for 82.54% and 97.83% of the raw sequences, respectively, and their average lengths were 237 and 216, respectively. As the number of sequences increases, so does the microbial diversity index. At the final stage, the dilution curve became flat, indicating that the sequencing data for this study reached saturation and could cover most microbial communities in rhizosphere soil (**Figures 3A–D**). Based on a 97% similarity score, 1007 OTUs were classified from the effective fungal sequences: 11 phyla, 173 families, 319 genera, and 521 species (**Figure 3E**). 54 OTUs were classified from the AMF effective sequences, including 1 phylum, 3 families, 3 genera, and 17 species (**Figure 3F**). The Sobs, Chao, and ACE indexes represent species richness indexes. The higher the index value, the more diverse the microbial community composition. The Shannon and Simpson indexes represent microbial diversity indexes. The higher the Shannon index or lower the Simpson index, the more diverse the microbial community composition. Inoculation with the PGPR significantly increased the richness of fungal community in wheat rhizosphere soil, decreased the diversity of AMF community, increased fungal diversity (*P*< 0.05), and had no significant effect on AMF diversity (**Table 4**).

**Table 4:** Diversity index of fungi and AMF in wheat rhizosphere soil samples under different treatments.

	Sample	sobs	shannon	simpson	ace	chao	coverage
Fungi	CK	531.60 ± 24.43b	3.42 ± 0.12b	0.09 ± 0.01a	658.75 ± 45.93a	666.88 ± 46.01a	0.997962b
	BIO	578.20 ± 17.31a	3.83 ± 0.05a	0.06 ± 0.01b	677.42 ± 27.74a	671.27 ± 18.28a	0.998104a
AMF	CK	37.60 ± 1.67a	2.58 ± 0.62a	0.16 ± 0.18a	37.90 ± 1.76a	37.60 ± 1.67a	0.999969a
	BIO	26.40 ± 2.88b	2.28 ± 0.46a	0.17 ± 0.11a	28.04 ± 3.45b	26.80 ± 3.03b	0.999938a

Data are means ± standard deviation (SD) (n = 5). CK and BIO represent the control and combined biocontrol agents inoculated plants, respectively. The small letters in the table represent the significant difference between the indexes of uninoculated and inoculated compound microbial agent wheat, P < 0.05.

**Figure 4:**
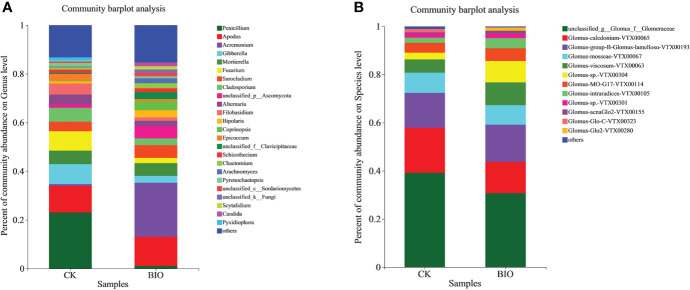
Relative abundance of fungal and AMF communities in wheat rhizosphere soil. **(A)** Relative abundance of fungal community in wheat rhizosphere soil at the genus level. **(B)** Relative abundance of AMF community in wheat rhizosphere soil at the species level. Different colors represent different species, and the rectangular area represents the percentage of species. CK and BIO represent the control and combined biocontrol agents inoculated plants, respectively.

In terms of fungi community information in the rhizosphere at the genus level. The relative abundance of *Apodus, Acremonium, Sarocladium, Coprinopsis, Schizothecium, Chaetomium* in the BIO treatment (12.01%, 22.15%, 5.24%, 3.65%, 1.66%, and 2.02%, respectively) was higher than that in the CK group (10.91%, 0.74%, 3.92%, 0.89%, 0.01%, 1.37%, and 0.91%, respectively). The relative abundance of *Penicillium*, *Gibberella*, *Mortierella*, *Fusarium*, *Cladosporium*, *Alternaria*, *Filobasidium* in the CK group (23.06%, 8.19%, 5.54%, 7.98%, 5.76%, 4.06%, and 4.53%, respectively) was higher than that in the BIO group (1.08%, 2.86%, 5.2%, 2.17%, 2.77%, 2.27%, and 1.41%, respectively). The relative abundance of *Arachnomyces* and *Candida* in the BIO group (2.10% and 1.55%, respectively) was higher than that in the CK group (both<0.01%). The relative abundance of *Epicoccum* and *Pyrenochaetopsis* in the CK group was 2.89% and 1.37%, respectively, but both accounted for<0.01% in the BIO group (**Figure 4A**). Regarding AMF community information in the wheat rhizosphere soil at the species level. The relative abundance of Glomus-group-B-Glomus-lamellosu-VTX00193, Glomus-viscosum-VTX00063, Glomus-sp.-VTX00304, Glomus-MO-G17-VTX00114, Glomus-intraradices-VTX00105, Glomus-acnaGlo2-VTX00155 in the BIO group (15.44%, 9.44%, 8.88%, 5.34%, 4.18%, and 1.36%, respectively) was higher than that in the CK group (14.55%, 5.47%, 2.81%, 4.10%, 2.12%, and 0.46%, respectively). The relative abundance of Glomus-mosseae-VTX000679, Glomus-caledonium-VTX00065, Glomus-sp.-VTX00301 in the CK group (8.40%, 18.65%, and 1.81%, respectively) was higher than that in BIO group (8.16%, 12.98%, and 1.75%, respectively). The relative abundance of Glomus-Glo2-VTX00280 in BIO group (1.38%) was higher than that in CK group (<0.01%). The relative abundance of Glomus-Glo-C-VTX00323 in CK group (1.42%) was higher than in the BIO group (< 0.01%) (**Figure 4B**).

**Figure 5:**
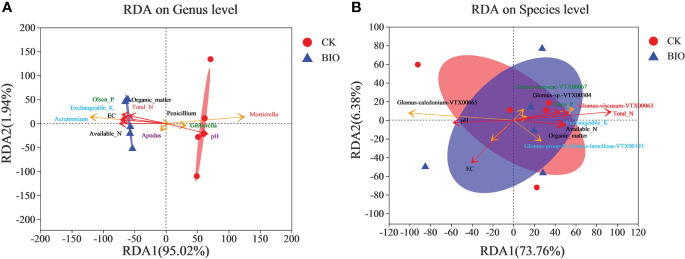
Redundancy Analysis (RDA) of AMF community based on Bray-Curtis distances. **(A)** fungal communities. **(B)** AMF communities. CK and BIO represent the control and combined biocontrol agents inoculated plants, respectively.

RDA results revealed that the fungal communities of different treatments were significantly separated, but AMF community was not significantly separated. EC, exchangeable K, available N, total N, organic matter, and Olsen P, primarily *Acremonium* and *Apodus*, were positively correlated with changes in the fungal community in wheat rhizosphere soil under CK group. A positive correlation was observed between pH and the change in the fungal community in BIO treatment, including *Gibberella* and *Mortierella*. *Penicillium* was less affected by the soil’s chemical properties (**Figure 5A**). The interaction between soil chemical properties and the AMF community revealed that Glomus-caledonium-VTX00065 was significantly positively correlated with pH and EC. Glomus-mosseae-VTX00067, Glomus-sp.-VTX00304, Glomus-viscosum-VTX00063, Glomus-group-B-Glomus-lamellosu-VTX00193 were positively correlated with exchangeable K, available N, total N, organic matter, and Olsen P (**Figure 5B**).

**Figure 6:**
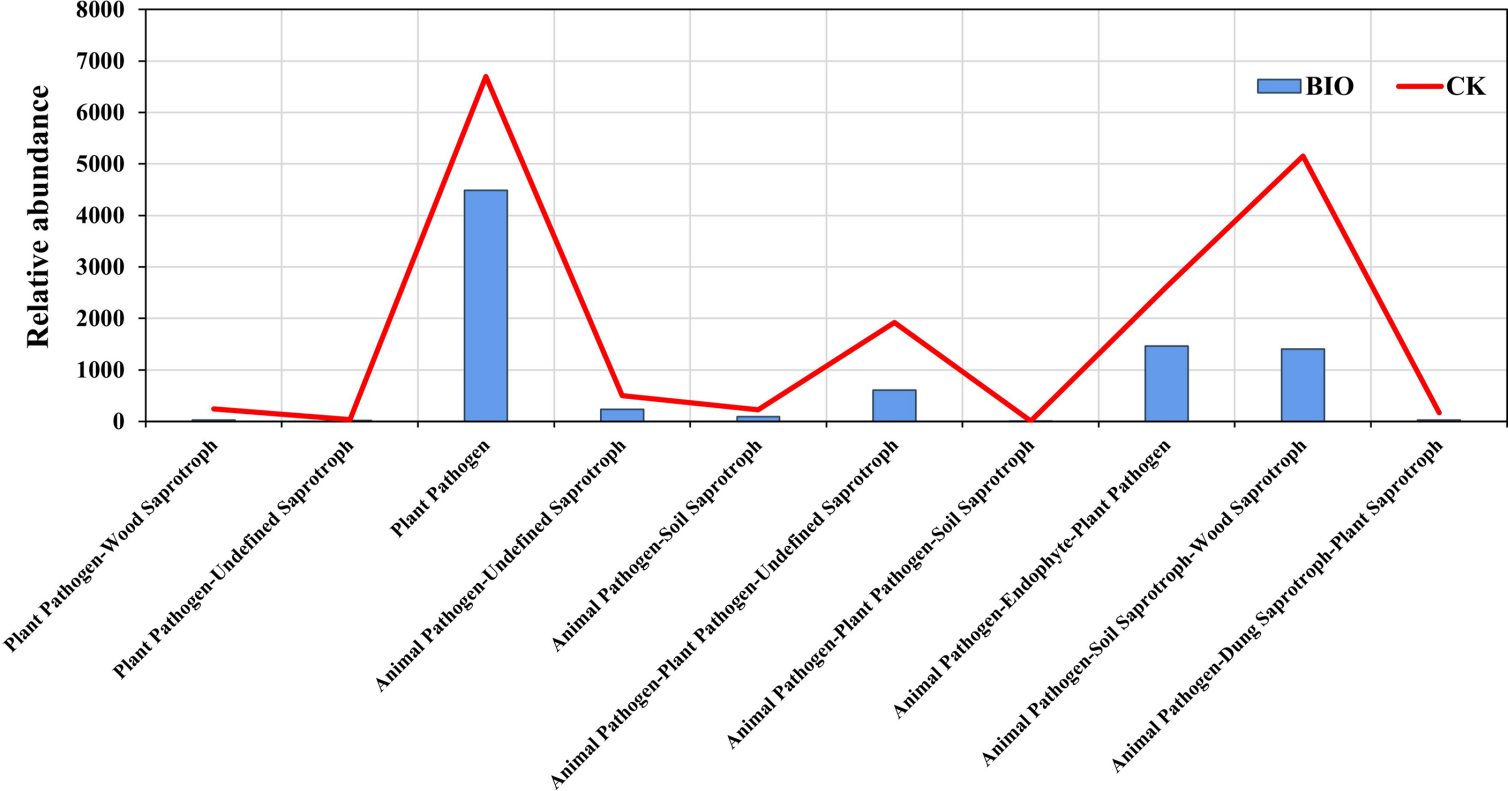
Variations in the composition of fungal functional groups inferred by FUNGuild. CK and BIO represent the control and combined biocontrol agents inoculated plants, respectively.

**Figure 7:**
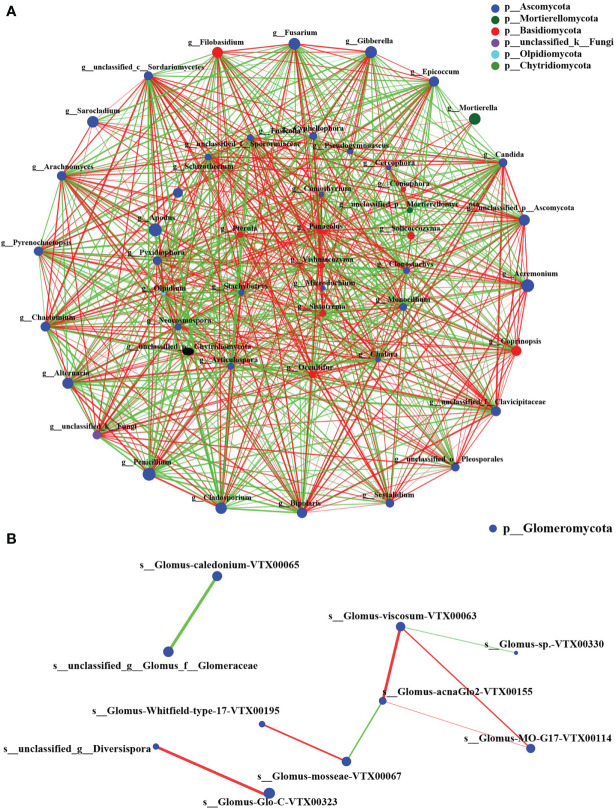
The co-occurrence network on virtual taxon level of fungi and AMF. **(A)** The genus-level fungal correlation. **(B)** The species level AMF correlation. The size of nodes in the figure represents the abundance of species, and different colors represent different species. The color of connection represents positive and negative correlation; red represents positive correlation; green represents negative correlation; the thickness of line indicates the size of correlation coefficient. The thicker the line, the higher the correlation between species. The more lines, the closer the relationship between the species and other species. Species with *P*< 0.05 are shown by default in the figure.

The fungal communities of two groups were classified and analyzed using a microecological guild. The relative abundance of plant pathogen, animal pathogen, and soil saprotroph functional guilds in wheat rhizosphere soil fungi in the CK group was higher than in the BIO group (**Figure 6**). The fungal genera directly related to the pathogen: *Gibberella*, *Cladosporium*, *Fusarium*, *Alternaria*, and *Bipolaris* (**Table S1**). The co-occurrence networks were used to explore the co-occurrence relationships between fungal genera and AMF species. The fungal collinear network contained five phyla: Ascomycota, Mortierellomycota, Basidiomycota, Olpidiomycota, and Chytridiomycota. There are 37 genera with more than 30 nodes, including *Panaeolus*, *Epicoccum*, *Coniothyrium*, and *Sistotrema*, *Gibberella* (**Figure 7A**). The AMF collinear network includes Glomeromycota. Glomus-MO-G17-VTX00114s, Glomus-acnaGlo2-VTX00155, and Glomus-viscosum-VTX00063 are important nodes (**Figure 7B**).

**Figure 8:**
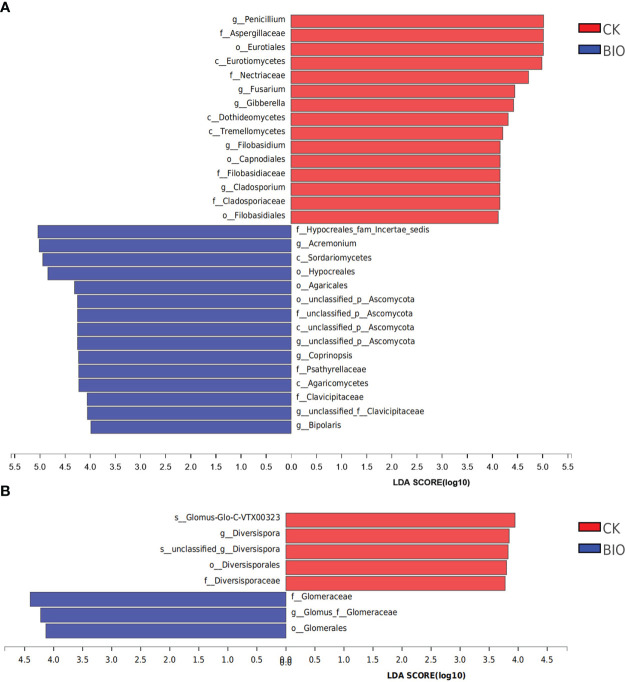
Discriminant analysis of multi-level species differences in CK and BIO groups. Indicator fungi and AMF with LDA scores of 3 or greater in communities associated with soil from CK and BIO groups. **(A)** fungal communities. **(B)** AMF communities. CK and BIO represent the control and combined biocontrol agents inoculated plants, respectively. Different-colored regions represent different constituents (red, CK; blue, BIO). The greater the LDA score, the greater the effect of species abundance on the difference. Class (c), Order (o), Family (f), Genus (g), Species (s).

The relative abundance of *Penicillium*, *Fusarium*, *Gibberella*, *Filobasidium*, and *Cladosporium* in the CK group was significantly higher than in the BIO group (*P*< 0.05). The relative abundance of *Acremonium*, *Coprinopsis*, and *Bipolaris* in the BIO group was significantly higher than that in the CK group (*P*< 0.05) (**Figure 8A**). The relative abundance of AMF species, such as Glomus-Glo-C-VTX00323, in the CK group was significantly higher than that in the BIO group (*P*< 0.05). The relative abundance of Glomus in the BIO group was significantly higher than that in the CK group (*P*< 0.05) (**Figure 8B**).

## Discussion

Agricultural development in ecologically fragile areas is hampered by geographical location, natural conditions, and other factors and is confronted with severe challenges of salt stress. The climatic conditions with a high evaporation-precipitation ratio and a scarcity of freshwater resources are prone to salinization, which is the main natural limiting factor for land use in most countries (Ashraf et al., 2018). Effectively improving the quality of crop rhizosphere soil can ensure improved wheat yield and quality under biotic and abiotic stresses (Simón et al., 2020). Non-salt-tolerant PGPRs are susceptible to synergistic metabolism and antagonism by indigenous microbial community in saline-alkaline soils, their ecological functions are limited, or they gradually lose colonization and plant growth promoting (PGP) properties with increasing salinity (Etesami and Beattie, 2018). Therefore, exploring microbial improvement measures that meet the requirements of soil quality improvement and health conservation in saline-alkali land is challenging and urgent.

Small molecular organic acids and siderophores secreted by salt-tolerant PGPR can decompose insoluble minerals *via* chelation, ion exchange, and acidification, thereby increasing the availability of nutrients in the soil, lowering pH, and alleviating Na^+^ stress in wheat (Delvasto et al., 2006; Chang and Yang, 2009; Saha et al., 2016; Ramakrishna et al., 2019). Both strains used in this study were salt-tolerant bacteria that can tolerate at least 12% (w/v) NaCl stress (Ji et al., 2021; Ji et al., 2022). This will greatly improve the strain’s ability to colonize in saline-alkali land and maintain high biological activity. The EC, exchangeable potassium, olsen P, total N, available N, and organic matter of wheat rhizosphere soil in BIO group were significantly higher than those in the CK group (*P*< 0.05) (**Table 1**). The finding demonstrates that both strains have competitive advantages in microbial-microbial interactions and can achieve mutual benefit in plant-microbial interactions. Similarly, previous studies have shown that strain salt tolerance and ACC deaminase-producing activity directly influence the effects of bacterial agents on saline agricultural fields. For example, under salt stress, ACCD-producing *Pseudomonas fluorescens* N3 and *Pseudomonas putida* Q7 strains increased maize root growth by 330% and plant height by 230%, respectively (Kausar and Shahzad, 2006). *Azospirillum brasilense* FP2 can reduce ACC oxidase expression and promote root growth in wheat plants (Camilios-Neto et al., 2014). Inoculation of salt-tolerant PGPB *Micrococcus yunnanensis*, *Planococcus rifietoensis*, and *Variovorax paradoxus* into sugar beet (*Beta vulgaris* L.) can increase seed germination and plant biomass, enhance photosynthesis, and significantly reduce stress-induced ethylene content (Zhou et al., 2017). Klebsiella, Pseudomonas, Agrobacterium, and Ochrobactrum strains isolated from the roots of *Arthrocnemum indicum* were *nifH*-positive and were able to produce indole-3 acetic acid (ranging from 11 to 19.1µg mL^-1^). In nutrient broth medium, all isolates showed tolerance to NaCl ranging from 4 to 8%. Under salt stress, inoculated peanut seedlings maintain ionic balance, accumulate less reactive oxygen species (ROS), and show enhanced growth compared to uninoculated seedlings (Sharma et al., 2016).

Inoculation of PGPR under salt stress improves the efficiency of plants to absorb selective ions by regulating the expression of ion transporter (High-affinity K^+^ Transporter, *HKT1*) to maintain a high K^+^/Na^+^ ratio, reduce the accumulation of Na^+^ and Cl^-^ ions, and regulate the balance of macronutrients and micronutrients in plants and soils (Islam et al., 2016; Etesami and Beattie, 2018). Inoculation with ACCD-producing *Bacillus licheniformis* HSW-16 can protect wheat plants from growth inhibition caused by NaCl and increase plant growth (6-38%) in terms of root length, shoot length, fresh weight, and dry weight. Ionic analysis of plant samples showed that bacterial inoculation decreases the accumulation of Na^+^ content (51%) and increases K^+^ (68%) and Ca^2+^ content (32%) in plants treated with different concentrations of NaCl (Singh and Jha., 2016). Additionally, AMF communities have been shown to induce sugar accumulation in plant roots further, leading to more organic acids being secreted into the soil, lowering plant ion toxicity, and enhancing antioxidant and detoxification defense systems (Abdelgawad et al., 2022). These effects may be the direct cause of higher EC in the BIO group than in the CK group (**Table 1**).

Photosynthesis is the basis of dry matter production and the driving force behind final yield. PGPR affects the expression of multiple proteins involved in plant photosynthesis, antioxidant processes, transmembrane transport, and pathogenesis (Cheng et al., 2012). In this study, the photosynthetic characteristics of wheat after flowering were significantly reduced on days 20 and 30 (*P*< 0.05), but the SPAD, *Pn*, and *WUE*_L_ of wheat in the BIO group were significantly higher than those in the CK group (*P*< 0.05) (**Figure 1**). This may be related to the characteristics that both strains can produce siderophores and proline. The siderophore can specifically and strongly bind Fe^3+^ to form an absorbable organic chelate. The enriched Fe is an integral part of key enzymes involved in plant respiration, photosynthesis, and other reactions (Kobayashi and Nishizawa, 2012). Osmoregulation substances such as proline can protect the structure and function of biological macromolecules, scavenge free radicals, and buffer the redox potential of cells to reduce enzyme inactivation caused by salt stress and ensure physiological activities such as photosynthesis (Ashraf and Foolad, 2007; Kohler et al., 2009). In this study, the grain number and yield of wheat in the BIO group were significantly higher than those in the CK group (**Table 3**). The main reason may be increased rhizosphere soil nutrients (Yadav et al., 2021). Concurrently, PGPR and AMF synergistically increase nutrient absorption, enhance photosynthesis, optimize rhizosphere bacteria, and improve soil health. The factors above can improve plant development, biofortification, and grain yield, consistent with previous studies (Smith and Sally, 2008; Cameron et al., 2013; Singh and Prasanna, 2020).

Wheat root rot and basal stem rot are soil-borne diseases caused by pathogens such as *Alternaria*, *Fusarium*, and *Gibberella*, which can cause seed decay and seedling death during wheat planting, basal stem browning at the adult stage, and white spike disease in severe cases, seriously affecting wheat yield and quality (Yuan et al., 2012; Gaju et al., 2014). To cope with climate change and increased abiotic stress in cultivated land, it is crucial to control disease occurrence and enhance crop stress resistance to achieve long-term sustainable agricultural development (Simón et al., 2021). Lowering the number of pathogens in soil by inoculating PGPR is an effective biological control measure (Ju et al., 2021; Vogel et al., 2021; Wang et al., 2022). As a major member of PGPR, *Bacillus* has a higher affinity for iron, which can deprive pathogenic microorganisms of nutrients and inhibit their pathogenesis (Radhakrishnan et al., 2017). *Bacillus amyloliquefaciens* subsp. *Plantarum* XH-9 has potential as a biocontrol agent when applied to local arable land to prevent the damage caused by *Fusarium oxysporum* and other phytopathogens (Wang et al., 2018). AMF not only competes for a niche with pathogens and produces mechanical defense barriers to reduce the number of pathogens such as *Fusarium* (Hao et al., 2010); AMF can also induce plants to produce protective enzymes and regulate root exudates/secondary metabolites, reduce root damage caused by pathogens, induce systemic defense systems in crops, and further enhance nutrient and water absorption (Geel et al., 2017). It is unclear how the application of antagonistic bacteria affects wheat rhizosphere fungi, especially AMF. In this study, diseased plants (79.24%) and disease incidence (79.84%) in the BIO group were significantly lower than those in the CK group (*P*< 0.05), and relative efficacy was 79.80% (**Table 2**). Our results showed that inoculation of antagonistic bacteria into the rhizosphere of plants could significantly alter the microbial community structure of arbuscular mycorrhizal fungi in the wheat rhizosphere (**Figure 4**), which may explain why plants produce ‘induced systemic tolerance’. Differences in fungal community function between the BIO and CK groups confirmed this result (**Figure 6**). All these findings demonstrate that combined *Bacillus* spp. inoculation can effectively control soil-borne diseases.

The biocontrol effect of microbial agents is easily affected by many biological and non-biological factors. In this study, salt stress and pathogens increased the complexity of PGPR biocontrol mechanisms in the field. Illumina MiSeq sequencing results revealed that microbial agents increased the richness and diversity of fungi (**Table 4**). The relative abundance of *Apodus*, *Acremonium*, *Sarocladium*, *Coprinopsis*, *Schizothecium*, and *Chaetomium* in the BIO group was higher than in the CK group. *Chaetomium* is widely used and studied because it produces a variety of secondary metabolites with antibacterial effects (Shanthiyaa et al., 2013). The relative abundance of *Penicillium*, *Gibberella*, *Mortierella*, *Fusarium*, *Cladosporium*, *Alternaria*, and *Filobasidium* in the CK group was higher than that in the BIO group (**Figures 4A**, **8A**). *Gibberella* and *Mortierella* were positively correlated with soil pH (**Figure 5A**), which may be attributed to the weakened inhibitory effect of combined *Bacillus* spp. inoculation at high pH. Although *Penicillium* has an antibacterial effect (Larena et al., 2018), its inhibitory effect on *Gibberella, Fusarium, Alternaria*, and other pathogens was not demonstrated in this study. We speculate that *Penicillium* is inhibited by the inoculated compound microbial agent or that it cannot produce enough antibacterial metabolites under salt stress.

Glomus is a major AMF fungus infecting plants (Landwehr et al., 2002). Previous studies have demonstrated that inoculation with Glomus helps stimulate plant growth and biomass (Campos-Soriano et al., 2010; Naseer et al., 2022). However, glomus’s mycelial growth is susceptible to salt stress (Juniper and Abbott, 2006). In this study, Glomus was the most common AMF genus in the CK and BIO groups (**Figure 4B**), but not among all fungi (**Figure 4A**). In the BIO group, Glomus-group-B-Glomus-lamellosu-VTX00193, Glomus-viscosum-VTX00063, Glomus-sp.-VTX00304, Glomus-MO-G17-VTX00114, Glomus-intraradices-VTX00105, Glomus-acnaGlo2-VTX00155, Glomus-Glo2-VTX00280 were the most common bacteria. Their relative abundance in the BIO group was higher than in the CK group (**Figure 4**). Glomus-group-B-Glomus-lamellosu-VTX00193, Glomus-viscosum-VTX00063 and Glomus-sp.-VTX00304 were positively correlated with organic matter, total N, exchangeable K, Olsen P (**Figure 5B**). Not only PGPR directly influenced AMF community structure and function, but also the changes in rhizosphere soil chemical properties have a selective effect on community composition. AMF has broad-spectrum benefits and potential application value under adverse conditions (Guo et al., 2022). The effect of AMF on plant growth was negatively correlated with the complexity of symbiotic network (Vannini et al., 2016; Pawlowska et al., 2018). In this study, AMF strains did not exhibit a complex symbiotic network (**Figure 7B**), indicating that AMF may strongly affect wheat. Current studies have confirmed that the combination of PGPR and AMF can exert greater growth-promoting characteristics to stimulate plant growth, which is better than inorganic fertilizer (Yadav et al., 2020; Yadav et al., 2021). Therefore, because of the universality of AMF and its potential impact on plant nutrient uptake and growth, it is crucial to verify the effect of biocontrol agents on AMF communities in the field.

## Conclusion

Our experiments established that a compound bacterial agent (consisting of *Bacillus subtilis* HG-15 and *Bacillus velezensis* JC-K3) improved wheat plant growth and yield under salt stress. They improved soil chemical properties, enhanced wheat photosynthesis, and *WUE*_L_, and significantly reduced wheat disease incidence. Illumina MiSeq sequencing results confirmed the inhibitory effect of combined *Bacillus* spp. inoculation on pathogens in wheat rhizosphere soil. Concurrently, the AMF community structure in the soil responded to combined *Bacillus* spp. inoculation, and multiple AMFs were correlated with soil chemical properties. This study deepens research on the ecological function of PGPR in the wheat rhizosphere in saline-alkali land. However, more research on the functions of different AMF strains and communities is required to fully understand the complexity and succession of the microbial community structure or the directional regulation of the AMF community.

## Data availability statement

The datasets presented in this study can be found in online repositories. The names of the repository/repositories and accession number(s) can be found in the article/**Supplementary Material**.

## Author contributions

CJ, ZC, and XK conceived the ideas and designed the experiment. CJ, ZC, XK, and ZX collected and analysed the samples. CJ, FS, CL, and JX analysed the data. CJ, ZC, KL, ZL, and HC prepared the figures and wrote the manuscript. CJ, ZL and HC revised and edited the draft. All authors made significant contributions to the draft and gave final approve for publication.

## Supplementary material

Supplementary Table 1 Normalized relative abundance of assigned fungal functional guilds with treatment
