# Supplementary material for: Biocontrol and plant growth promotion by combined Bacillus spp. inoculation affecting pathogen and AMF communities in the wheat rhizosphere at low salt stress conditions
Source: Front Plant Sci. 2022 Dec 8;13:1043171. doi: 10.3389/fpls.2022.1043171 (PMC9773258; doi:10.3389/fpls.2022.1043171)
Supplement: Supplementary Table 1 — Normalized relative abundance of assigned fungal functional guilds with treatment [file Table_1.doc]

Table S1 | Normalized relative abundance of assigned fungal functional guilds with treatment

| Guild | BIO | CK |
| --- | --- | --- |
| Plant Pathogen-Wood Saprotroph | 21.6 | 240.6 |
| Plant Pathogen-Undefined Saprotroph | 14.8 | 32.2 |
| Plant Pathogen | 4484.6 | 6694.6 |
| Animal Pathogen-Undefined Saprotroph | 236 | 498.6 |
| Animal Pathogen-Soil Saprotroph | 90 | 227 |
| Animal Pathogen-Plant Pathogen-Undefined Saprotroph | 604.6 | 1921.2 |
| Animal Pathogen-Plant Pathogen-Soil Saprotroph | 3.8 | 4.4 |
| Animal Pathogen-Endophyte-Plant Pathogen | 1464.2 | 2619.8 |
| Animal Pathogen-Soil Saprotroph-Wood Saprotroph | 1404.4 | 5155.4 |
| Animal Pathogen-Dung Saprotroph-Plant Saprotroph | 23.4 | 168 |

CK and BIO represent the control and combined biocontrol agents inoculated plants, respectively.
